# A Prognostic Model for Breast Cancer With Liver Metastasis

**DOI:** 10.3389/fonc.2020.01342

**Published:** 2020-09-02

**Authors:** Lei Ji, Lei Fan, Xiuzhi Zhu, Yu Gao, Zhonghua Wang

**Affiliations:** ^1^Department of Medical Oncology, Fudan University Shanghai Cancer Center, Shanghai Medical College, Fudan University, Shanghai, China; ^2^Department of Oncology, Shanghai Medical College, Fudan University, Shanghai, China; ^3^Department of Breast Surgery, Fudan University Shanghai Cancer Center, Shanghai Medical College, Fudan University, Shanghai, China

**Keywords:** breast cancer, liver metastasis, prognostic factors, prognostic model, prognostic score

## Abstract

**Background:** Breast cancer with liver metastasis consists of a group of heterogeneous diseases, and survival time may be significantly different, ranging from a few months to several years. The present study aimed to develop and externally validate a prognostic model for breast cancer with liver metastasis (BCLM).

**Methods:** In total, 1022 eligible patients from January 2007 to December 2018 were selected from Fudan University Shanghai Cancer Center (FUSCC) and were temporally in the training (*n* = 715) and validation (*n* = 307) set. According to regression coefficients found in the multivariate Cox regression analysis, the final results were transformed into the prognostic scores. On the basis of these scores, patients were finally classified into three risk groups, including low-, intermediate-, and high-risk groups. Bootstrapping was used for internal validation. Then, time-dependent receiver operating characteristic (ROC) curves and calibration plots were used to assess discrimination and calibration of this prognostic model in the validation set.

**Results:** Molecular subtypes, metastatic-free interval (MFI), extrahepatic metastasis, and liver function tests were identified as independent prognostic factors in the multivariate analysis. According to risk stratification, intermediate-risk (hazard ratio (HR) 2.12, 95% confidence interval (CI) 1.74–2.58, *P* < 0.001) and high-risk groups (HR 6.94, 95% CI 5.25–9.16, *P* < 0.001) had significantly worse prognoses in comparison with the low-risk group regarding overall survival (OS) from the time of metastasis. The median OS in these three groups were 39.97, 21.03, and 8.80 months, respectively. These results were confirmed in the internal and external validation cohorts.

**Conclusions:** Based on molecular classification of tumors, routine laboratory tests, and other clinical information easily accessible in daily clinical practice, we developed a clinical tool for BCLM patients to predict their prognosis. Moreover, it may be useful for identifying the subgroup with unfavorable prognosis and individualization of treatment.

## Introduction

About 25% of early breast cancer patients still experience local recurrence and develop distant metastases after active treatment (1), and nearly 10% of patients are in stage IV disease at initial diagnosis in the developed world, which is referred to as *de novo* metastatic breast cancer (MBC) (2). Despite treatment advances, MBC remains incurable, and up to 70–80% of MBC patients die of cancer within 5 years (2). As a result of the heterogeneity of MBC, multiple prognostic models based on prognostic factors or scores are developed to predict their clinical outcomes and direct clinicians to make appropriate therapeutic decisions (3–5). Furthermore, there are also a variety of prognostic models for breast cancer with brain metastasis (BCBM) for clinicians to accurately predict survival in these patients (6, 7).

Breast cancer can metastasize anywhere in the body but primarily metastasizes to the lung, bone, liver, non-axillary lymph nodes, and brain (8). Notably, liver metastasis (LM) is not only a common metastatic site but also associated with significantly increased death risk similar to brain metastasis, a disproportionally higher mortality compared with lung or bone metastases (5, 8). As graded prognostic assessment (GPA) is widely accepted in BCBM patients, the abovementioned prognostic models stimulate us to propose a practical model for BCLM (3–7). Our aim was to construct a reliable and user-friendly prognostic model for BCLM and to evaluate its concordance and accuracy by validating it both internally and externally. The prognostic model might help clinicians to estimate death risk of BCLM patients and select optimal treatment.

## Patients and Methods

### Selection of Patients

The present study was approved by the independent ethics committees of FUSCC. Consecutive MBC patients diagnosed at FUSCC from January 2007 to December 2018 were identified (*n* = 9,062). Eligibility criteria were as follows: (1) histologically confirmed breast cancer, (2) female patients, (3) the liver as the first site of metastatic disease in either *de novo* metastatic or recurrent breast cancer, and (4) complete and detailed records. Patients with bilateral breast cancer, other invasive neoplasms, or having unknown follow-up were excluded. Finally, there were 1,022 eligible patients selected for further analysis. The full steps of selection are shown in Figure S1.

### Studying Variables and Selection of Cutoff Values

Information was collected on the following clinicopathologic variables: age, molecular subtypes, prior treatment information (surgery and systemic chemotherapy), MFI, sites of extrahepatic metastasis (brain, lung, bone, and lymph nodes), characteristics of liver metastasis (distribution, number, maximum diameter), hemoglobin (HB), liver function tests, and survival time. Molecular subtypes included hormone receptor (HR) and human epidermal growth factor receptor 2 (HER2) status. We defined MFI as the interval between the date of diagnosis of primary breast cancer and the date of first distant metastasis. The cutoff points of MFI used in this study were 3 and 24 months according to reference (9). Hence, patients could be divided into three groups: patients with *de novo* MBC (MFI < 3 months), patients with a short MFI (≤ 24 months), and patients with a long MFI > 24 months. Distant lymph node metastasis was defined as metastasis out of ipsilateral axillary, supraclavicular, infraclavicular, or internal mammary lymph nodes. The diagnosis of LM was based on the radiographic imaging or pathological evidence. Characteristics of liver metastasis were evaluated with the help of abdominal computed tomography (CT), magnetic resonance imaging (MRI), or surgical resection specimens. Liver function tests were also analyzed in the present study, including total bilirubin, alanine aminotransferase (ALT), aspartate aminotransferase (AST), alkaline phosphatase (ALP), lactate dehydrogenase (LDH), and γ-glutamyl transferase to albumin ratio (GAR). Blood samples were taken for laboratory tests within 1 week after the diagnosis of LM. Classifications of distribution, number, or maximum diameter of liver metastases and LDH were based on other studies (10–13). The cutoff values for HB, total bilirubin, ALT, AST, and ALP were determined according to the Common Terminology Criteria for Adverse Events (CTCAE) because moderate or grade II abnormalities in hemoglobin levels or liver function might influence therapeutic choices (14). The value of GAR was defined as a simple ratio between the serumγ-glutamyl transferase (GGT, U/L) level and the serum albumin (ALB g/L) level. OS from the time of metastasis was measured from the date of diagnosis of LM to death for any cause. The last follow-up time period was November 2019 with a median follow-up time of 18.63 months. If patients were lost to follow-up, it was censored on the last day of follow-up.

### Statistical Analysis

Patients were divided into the training (2007–2016, *n* = 715) and validation (2016–2018, *n* = 307) set at a ratio of 7:3. The optimal cutoff value for GAR was chosen by the calculation of the Youden index and receiver operating characteristic curve (ROC) analysis (Figure S2). Pearson chi-squared test or Fisher's exact test was utilized to make a comparison among categorical variables. Cox proportional hazards regression models with the backward selection method were used for multivariate analysis and to compute hazard ratios. The regression coefficients were calculated through multivariate regression analysis and then multiplied by 10 and rounded to represent final prognostic scores. Bootstrapping was used for internal validation. Then, time-dependent ROC curves and calibration plots were used to assess discrimination and calibration of this prognostic model in the validation set. The cutoff values of risk groups were determined by X-tile plots (15). Survival analysis was performed using Kaplan-Meier survival curves, and log-rank tests were used to compare survival curves. All *P*-values were two-sided, and values of *P* < 0.05 were considered statistically significant. Statistical analysis was carried out using SPSS software (SPSS 20, Chicago, IL, USA) and R software version 4.0.1.

## Results

### Characteristics and Relationship With Risk Stratification

A total of 1,022 patients selected in this study were divided into training (*n* = 715) and validation (*n* = 307) sets. A description of clinicopathologic characteristics is given in Table 1 and Table S1. In the training group, there were 126 patients (17.6%) diagnosed with *de novo* MBC and 589 patients (82.4%) with recurrent MBC, of which 297 patients (50.4%) had a short MFI and 292 patients (49.6%) had a long MFI. Median age at the diagnosis of LM was 50 (range 21–87) years with 617 patients (86.3%) younger than 60 years. Of these patients, the proportion of HR-positive and HER2-positive patients was 62.2 and 38.0%, respectively. Among recurrent MBC patients, almost all patients underwent surgery on the primary tumor (584, 99.2%) and received (neo)adjuvant chemotherapy (569, 96.6%). Patterns of distant metastasis showed that the most common site of extrahepatic metastasis was bone (42.7%) followed by distant lymph nodes (36.1%), lung (25.6%), and brain (2.9%). Liver metastasis was characterized by diffuse distribution and small nodules, the majority of which were multiple (≥3 metastases, 77.3%) but small nodules (≤3 cm, 59.4%) and involved right and left lobes (74.4%). In the early stage of LM, liver function was impaired to varying degrees but generally mild. The rise of LDH (36.1%) and GAR (35.9%) seemed to be sensitive liver dysfunction indicators, and moderate or above abnormalities in HB (2.9%), total bilirubin (2.7%), ALT (3.6%), AST (9.5%), and ALP (3.9%) levels were uncommon according to CTCAE. Compared with the low-risk group, the intermediate- and high-risk group presented with a higher rate of HR and HER2 negativity, a short MFI, extrahepatic metastasis, liver metastasis tumor load, and abnormalities in hemoglobin levels or liver function (all *P*s ≤ 0.001), suggesting that these factors might affect the prognosis of BCLM.

**Table 1:** Baseline characteristics and its relationship with risk stratification of training set.

	**Score**				**Sig**
	**Total *N*= 715, (%)**	**Low risk *N* = 341, (%)**	**Intermediate risk *N* = 286, (%)**	**High risk *N* = 88, (%)**	
**Age, years**					
≤60	617 (86.3)	291 (85.3)	253 (88.5)	73 (83.0)	0.328
>60	98 (13.7)	50 (14.7)	33 (11.5)	15 (17.0)	
**HR status**					
Negative	270 (37.8)	75 (22.0)	123 (43.0)	72 (81.8)	<0.001
Positive	445 (62.2)	266 (78.0)	163 (57.0)	16 (18.2)	
**HER2 status**					
Negative	443 (62.0)	176 (51.6)	202 (70.6)	65 (73.9)	<0.001
Positive	272 (38.0)	165 (48.4)	84 (29.4)	23 (26.1)	
**Surgery of primary site**					
No	131 (18.3)	102 (29.9)	24 (8.4)	5 (5.7)	<0.001
Yes	584 (81.7)	239 (70.1)	262 (91.6)	83 (94.3)	
**Prior (neo)adjuvant chemotherapy**					
No	146 (20.4)	109 (32.0)	26 (9.1)	11 (12.5)	<0.001
Yes	569 (79.6)	232 (68.0)	260 (90.9)	77 (87.5)	
**MFI, months**					
*De novo* metastatic	126 (17.6)	103 (30.2)	20 (7.0)	3 (3.4)	<0.001
MFI≤24	297 (41.5)	89 (26.1)	144 (50.3)	64 (72.7)	
MFI>24	292 (40.8)	149 (43.7)	122 (42.7)	21 (23.9)	
**Brain metastases**					
No	694 (97.1)	341 (100.0)	278 (97.2)	75 (85.2)	<0.001
Yes	21 (2.9)	0	8 (2.8)	13 (14.8)	
**Lung metastases**					
No	532 (74.4)	288 (84.5)	195 (68.2)	49 (55.7)	0.001
Yes	183 (25.6)	53 (15.5)	91 (31.8)	39 (44.3)	
**Bone metastases**					
No	410 (57.3)	250 (73.3)	133 (46.5)	27 (30.7)	<0.001
Yes	305 (42.7)	91 (26.7)	153 (53.5)	61 (69.3)	
**Distant lymph nodes metastases**					
No	457 (63.9)	241 (70.7)	180 (62.9)	36 (40.9)	<0.001
Yes	258 (36.1)	100 (29.3)	106 (37.1)	52 (59.1)	
**Liver metastases distribution**					
Unilobar	183 (25.6)	114 (33.4)	59 (20.6)	10 (11.4)	<0.001
Bilobar	532 (74.4)	227 (66.6)	227 (79.4)	78 (88.6)	
**Number of liver metastases, No**					
1 or 2	162 (22.7)	102 (29.9)	51 (17.8)	9 (10.2)	<0.001
≥3	553 (77.3)	239 (70.1)	235 (82.2)	79 (89.8)	
**Maximum diameter of liver metastases, cm**					
≤3	425 (59.4)	241 (70.7)	143 (50.0)	41 (46.6)	<0.001
>3	290 (40.6)	100 (29.3)	143 (50.0)	47 (53.4)	
**Hb, g/L**					
<100	21 (2.9)	6 (1.8)	6 (2.1)	9 (10.2)	0.001
≥100	694 (97.1)	335 (98.2)	280 (97.9)	79 (89.8)	
**Total bilirubin**					
≤1.5 ULN	696 (97.3)	339 (99.4)	282 (98.6)	75 (85.2)	<0.001
>1.5 ULN	19 (2.7)	2 (0.6)	4 (1.4)	13 (14.8)	
**ALT**					
≤3 ULN	689 (96.4)	339 (99.4)	272 (95.1)	78 (88.6)	<0.001
>3 ULN	26 (3.6)	2 (0.6)	14 (4.9)	10 (11.4)	
**AST**					
≤3 ULN	647 (90.5)	328 (96.2)	253 (88.5)	66 (75.0)	<0.001
>3 ULN	68 (9.5)	13 (3.8)	33 (11.5)	22 (25.0)	
**LDH, U/L**					
≤250	457 (63.9)	327 (85.60)	123 (48.24)	7 (8.97)	<0.001
>250	258 (36.1)	55 (14.40)	132 (51.76)	71 (91.03)	
**ALP**					
≤2.5 ULN	687 (96.1)	337 (98.8)	272 (95.1)	78 (88.6)	<0.001
>2.5 ULN	28 (3.9)	4 (1.2)	14 (4.9)	10 (11.4)	
**GAR**					
≤1.5	458 (64.1)	302 (88.6)	144 (50.3)	12 (13.6)	<0.001
>1.5	257 (35.9)	39 (11.4)	142 (49.7)	76 (86.4)	

*HR, hormone receptor; HER2, human epidermal growth factor receptor 2; MFI, metastasis-free interval; HB, hemoglobin; ULN, upper limits of normal; ALT, alanine aminotransferase; AST, aspartate aminotransferase; LDH, lactate dehydrogenase; ALP, alkaline phosphatase; GAR, γ-glutamyl transferase to albumin ratio*.

### Prognostic Model and Validation

**Figure 1:**
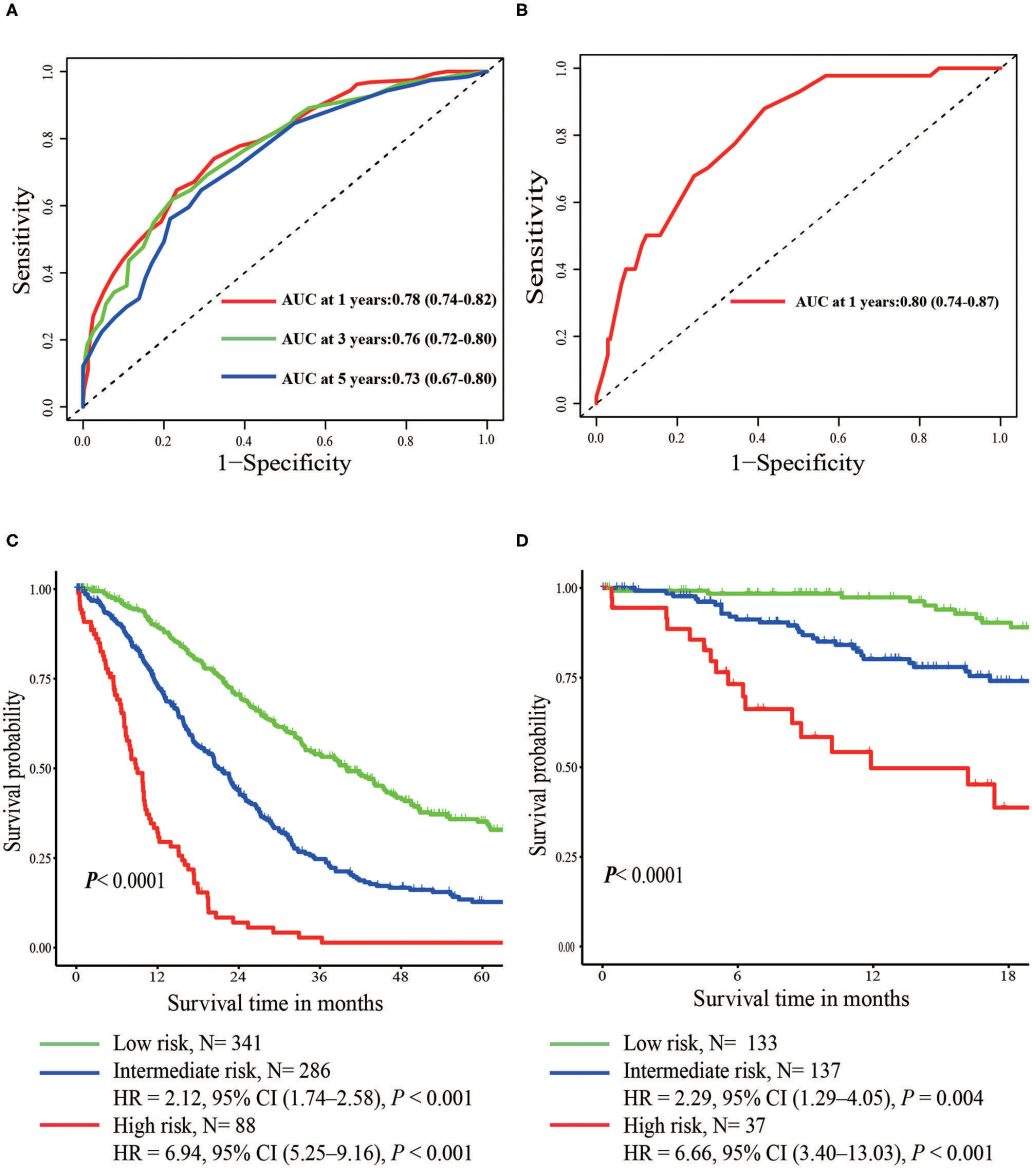
Time-dependent ROC curves of the prognostic model in the training set **(A)** and the validation set **(B)**. Overall survival from metastasis of the three prognostic groups in the training **(C)** and validation set **(D)**.

**Figure 2:**
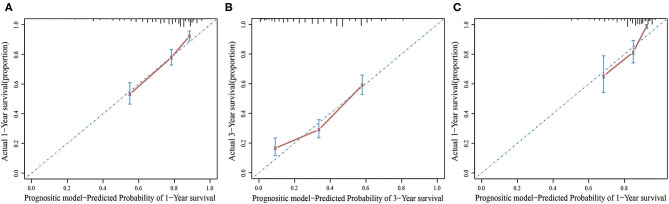
The calibration curve for predicting patient survival at 1 year **(A)** and 3 years **(B)** in the training set and at 1 year **(C)** in the validation set.

In the multivariate Cox regression model, molecular subtypes (HR and HER2 status), MFI, sites of extrahepatic metastasis (brain, lung, and bone metastasis), and liver function tests (total bilirubin, LDH, and GAR) were associated with OS (Table 2). Specifically, the death risk of HR negative (HR 1.740, 95% CI 1.424–2.127, *P* < 0.001) and HER2 negative (HR 1.615, 95% CI 1.316–1.983, *P* < 0.001) patients increased compared to HR or HER2 positive counterparts. The length of MFI also had a significant impact on the survival of BCLM patients, and therefore, the survival of patients with a long MFI (HR 1.612, 95% CI 1.195–2.174, *P* < 0.001) was shortened in comparison with patients with *de novo* MBC, and patients with a short MFI (HR 2.563, 95% CI 1.904–3.449, *P* < 0.001) had more than twice the risk of death relative to them. In addition, presence of extrahepatic metastasis (brain, lung, and bone metastasis) and abnormal liver function (total bilirubin, LDH, and GAR) were all correlated with an unfavorable prognostic impact on OS. This prognostic model had the area under the curve (AUC) of time-dependent ROC at 1 year OS with 0.78 in the training set and 0.80 in the validation set, indicating this model had a good discrimination (Figure 1). The bootstrapping method was used to confirm the stability of our prognostic model in the training set, and the final results were rather robust (Table S2). Calibration curves of the prognostic model for 1 year and 3 year OS in the training set as well as 1 year OS in the validation set showed its good concordance (Figure 2).

**Table 2:** Multivariate Cox regression model (Training set).

	**Variables in the equation**						
	**B**	**SE**	**Wald**	**df**	**Significance**	**Exp(B)**	**95% CI for Exp(B)**
HR	0.554	0.102	29.235	1	0.000	1.740	1.424–2.127
HER2	0.480	0.105	21.043	1	0.000	1.615	1.316–1.983
MFI ≤ 24 (vs. *de novo*)	0.941	0.152	38.544	1	0.000	2.563	1.904–3.449
MFI > 24 (vs. *S de novo*)	0.477	0.153	9.786	1	0.002	1.612	1.195–2.174
Brain metastases	0.569	0.269	4.483	1	0.034	1.766	1.043–2.990
Lung metastases	0.270	0.104	6.777	1	0.009	1.310	1.069–1.606
Bone metastases	0.258	0.096	7.225	1	0.007	1.294	1.072–1.562
Total bilirubin	0.833	0.287	8.440	1	0.004	2.300	1.311–4.035
LDH	0.505	0.108	21.670	1	0.000	1.657	1.339–2.049
GAR	0.370	0.109	11.449	1	0.001	1.447	1.168–1.793

*B, regression coefficient; SE, standard error; Wald, test statistic; df, degrees of freedom; Exp(B), hazard ratio; HR, hormone receptor; HER2, human epidermal growth factor receptor 2; MFI, metastasis-free interval; LDH, lactate dehydrogenase; GAR, γ-glutamyl transferase-to-albumin ratio*.

### Risk Stratification and Survival

According to prognostic scores derived from regression coefficients, patients were stratified into three risk groups (Table 3). X-tile software was adopted to determine the optimal cutoff value of risk stratification, including low- (<16 points), intermediate- (16–25 points), and high-risk groups (>25 points). Intermediate- (HR 2.12, 95% CI 1.74–2.58, *P* < 0.001) and high-risk groups (HR 6.94; 95% CI 5.25–9.16; *P* < 0.001) had significantly worse prognosis in comparison with the low-risk group (Figure 1). In the validation set of 307 patients, intermediate- (HR 2.29, 95% CI 1.29–4.05, *P* = 0.004) and high-risk groups (HR 6.66, 95% CI 3.40–13.03, *P* < 0.001) were also related with significantly increased death risk compared with the low-risk group, showing the strong ability for discrimination in our prognostic model (Figure 1). The median OS of three groups were 39.97, 21.03, and 8.80 months with the 3 year OS rates of 54, 25, and 1%, respectively.

**Table 3:** Calculation of the score and cutoff points of the prognostic groups.

**Parameter**	**Value**	**Points**		
HR	Negative	6		
HER2	Negative	5		
MFI ≤ 24	≤24	9		
MFI > 24	>24	5		
Brain metastases	Yes	6		
Lung metastases	Yes	3		
Bone metastases	Yes	3		
Total bilirubin	>1.5 ULN	8		
LDH	>250 U/L	5		
GAR	>1.5	4		
For all other values		0		
Points	Prognostic groups	2-year survival	3-year survival	Median overall survival, months
<16	Low risk	70%	54%	39.97 (33.59–46.35)
16–25	Intermediate risk	44%	25%	21.03 (18.44–23.62)
>25	High risk	6%	1%	8.80 (6.78–10.82)

*HR, hormone receptor; HER2, human epidermal growth factor receptor 2; MFI: metastasis-free interval; LDH, lactate dehydrogenase; GAR, γ-glutamyl transferase-to-albumin ratio*.

## Discussion

In the present analysis of 1,022 patients treated in our institution from 2007 to 2018, we have shown that molecular subtypes (HR and HER2 status), MFI, sites of extrahepatic metastasis (brain, lung, and bone metastasis), liver function tests (total bilirubin, LDH, and GAR) were independent prognostic factors of BCLM patients. Hence, a prognostic model for BCLM patients was constructed using prognostic factors.

With regard to molecular subtypes, HR positive can indicate that breast cancer growth and proliferation are still regulated by hormones, called hormone-dependent tumors. At the same time, endocrine therapy has benefits including few adverse reactions and long-lasting effects, which can often bring long-term survival benefits to HR-positive patients. Therefore, HR positive has been regarded as a significant factor suggesting a favorable prognosis in early and metastatic breast cancer (1, 3–5, 8–10, 16). Although HER2 amplification and overexpression is considered to be a predictor of a risk of distant metastasis and breast cancer–related death, subsequently developed anti-HER2 agents have remarkably improved survival of HER2-positive breast cancer patients (2, 9, 17). In the era of targeted therapy, a study in southeastern Netherlands found that the mortality risk of HER2-negative patients was increased by 44% (95% CI 1.13–1.83, *P* = 0.003) compared with HER2 positive patients (9). In our study, the death risk of HR-negative and HER2-negative patients increased compared to HR or HER2-positive counterparts, consistent with these observations. Patients with a short MFI, usually defined as ≤24 months, had a worse prognosis (3, 5, 9). Furthermore, *de novo* MBC patients seemed to have a significantly better prognosis relative to recurrent MBC patients although this difference was gradually reduced with the extension of disease-free interval (DFI) or MFI (9, 18, 19). Lobbezoo et al. found that patients with MFI ≤24 months had significantly shorter survival time (9.1 vs. 29.4 months, *P* < 0.001) than those with *de novo* stage IV diseases, but patients with a longer MFI (>24 months) had a similar prognosis (27.9 vs. 29.4 months, *P* = 0.73) (9). The possible reason for this phenomenon is that patients with relapsed MBC previously undergoing systematic therapy may be more resistant to chemotherapy than therapy-naive patients with *de novo* MBC, but further investigation is warranted (9, 18, 19). This study confirmed that the survival time of patients with a long MFI was shortened in comparison with *de novo* MBC with patients with a short MFI conferring a significantly worse outcome.

MBC survival differed significantly according to metastatic sites and patients with visceral metastasis were associated with an increased risk of mortality compared with those with non-visceral metastasis (3–5, 9, 18). Moreover, the presence of brain metastasis had a larger impact on survival than any other metastatic sites (3, 5, 9, 18). It was not surprising that multiple metastases had a more unfavorable influence on the prognosis (3, 5, 9, 17, 18). In this analysis, extrahepatic metastasis, except for distant lymph nodes metastasis, could lead to shortened survival, probably because liver metastasis was the critically life-threatening factor compared with lymph node metastasis.

It should be noted that patients with higher bilirubin levels had much lower risk for several diseases in part via its antioxidant and anti-inflammatory properties (20). A recent study also reported that a nearly 40% reduction of the death risk was shown among early breast cancer patients with higher total bilirubin level (21). However, hyperbilirubinemia was associated with poor prognosis among BCLM patients (22). It is possible that, unlike relatively higher total bilirubin among non-metastatic breast cancer patients, a raised bilirubin 1.5 times more than the upper limit of normal (ULN) among BCLM patients may predict severe liver injury and, thus, contribute to drug discontinuation or hepatic encephalopathy.

The Warburg effect drives cancer cells to depend on aerobic glycolysis even when the oxygen supply is sufficient, which is the hallmark of cancer metabolism in contrast to normal tissues (23). LDH plays an indispensable role in glycolysis due to its ability of converting pyruvate to lactate during anaerobic conditions (24). Deregulated levels of LDH could reflect higher tumor burden, poorer treatment response, and prognosis, which had been previously reported in multiple tumors (21, 24–26). A meta-analysis on prognostic effect of LDH in breast cancer patients showed that higher LDH levels resulted in unsatisfactory OS and progression-free survival, which included 11 studies involving 6,102 patients (27).

GGT, the major endogenous antioxidant, makes precursor amino acids assimilated and involves in glutathione synthesis (28). GGT in tumors enables cells to quickly replenish glutathione after receiving pro-oxidant anticancer therapy and, therefore, is correlated with drug resistance and poor survival (29). Interestingly, Fentiman et al. reported that a significantly positive relationship was identified between elevated GGT and breast cancer risk (30). ALB synthesized by the liver functions as an antioxidant, transporter of nutrients, and has participation in signal pathways owing to its unique structural properties (31). Hypoalbuminemia usually occurs in advanced cancer patients and is attributed to various mechanisms, including impaired liver synthesis, increased catabolism, and cachexia (21, 31). Hypoalbuminemia is an independent indicator of poor prognosis of various tumors (21, 22, 32, 33). In contrast to decreased serum albumin levels, serum GGT levels tend to be elevated when liver function is impaired. Therefore, GAR based on the above two parameters may have the advantage to reflect liver reserve ability and predict the prognosis (32, 33). In the current study, abnormal liver function (total bilirubin, LDH, and GAR) were all correlated with an unfavorable prognostic impact on OS.

After analyzing the prognosis of 123 BCLM patients, Duan et al. found that patients with three or more liver metastases carried 2.26 times increased risk of death relative to patients with <3 liver metastases in multivariate analysis (10). However, characteristics of liver metastasis in this study appeared to make no difference. There are two potential reasons contributing to this discrepancy. First of all, the distribution, number, and maximum diameter of liver metastases were significantly different in the three risk groups, indicating that the risk of mortality increased with the expansion of liver metastasis in univariate analysis. Afterward, abnormal liver function was closely related to the tumor load of liver metastasis, adjusting the role of these factors in multivariate analysis.

There have been many studies on prognostic factors in the setting of BCLM. An analysis of 145 BCLM patients showed that hypoalbuminemia, older age, and ER negativity were independent predictors of poor survival (22). A Greek registry analysis obtained similar results, in which HR positivity, low histological grade, absence of extrahepatic metastasis, and good performance status were significant prognostic factors for favorable prognosis in univariate analysis (34). A recent population-based study including more than 4,000 patients with *de novo* BCLM has identified demographic and socioeconomic factors, pathological grade, total number of extrahepatic metastasis, treatment, and molecular subtype as parameters influencing overall survival significantly (17). Regierer et al. developed a prognostic score for MBC based on HR status, MFI, and sites of metastases to predict their prognosis and individualize optimal treatment, which was validated internally and externally (5). Unfortunately, HER2 status was not included in the prognostic score and, therefore, limited its applicability. The modified breast-GPA for BCBM patients integrating four simple clinical parameters had an immediate role in predicting their survival, but was not applicable for BCLM patients (7). To our knowledge, there is no prognostic model that integrates these prognostic factors for BCLM patients, so our model makes pragmatic sense.

There were also some limitations in this research. First, a single-center and retrospective study inevitably resulted in selection bias although our prognostic model was validated internally and externally. Second, patients who developed liver metastasis later in the disease course were excluded so that we were unable to evaluate these patients. Third, detailed information on performance status and treatment was not available in this study.

## Conclusions

Despite these limitations, this research can provide a simple and reliable prognostic model. The major advantage of our study is the incorporation of parameters easily accessible in clinical practice on the basis of the large sample of BCLM patients. Aside from exquisite classification of tumors according to next-generation sequencing methods, our model may help identify subgroups with different prognosis and guide subsequent therapy.

## Data Availability Statement

The original contributions presented in the study are included in the article/Supplementary Material, further inquiries can be directed to the corresponding author/s.

## Ethics Statement

The present study was approved by the independent ethics committees of FUSCC. The patients/participants provided their written informed consent to participate in this study.

## Author Contributions

LJ proposed the study. XZ and YG collected the data. LJ and LF analyzed the data and wrote the first draft. ZW critically reviewed the manuscript. All authors contributed to the article and approved the submitted version.

## Conflict of Interest

The authors declare that the research was conducted in the absence of any commercial or financial relationships that could be construed as a potential conflict of interest.

## Abbreviations

BCLM: breast cancer with liver metastasis
FUSCC: Fudan University Shanghai Cancer Center
ROC: receiver operating characteristic curve
MFI: metastatic-free interval
HR: hazard ratio
CI: confidence interval
OS: overall survival
MBC: metastatic breast cancer
BCBM: breast cancer with brain metastasis
LM: liver metastasis
GPA: graded prognostic assessment
HB: hemoglobin
HR: hormone receptor
HER2: human epidermal growth factor receptor 2
CT: computerized tomography
MRI: magnetic resonance imaging
ALT: alanine aminotransferase
AST: aspartate aminotransferase
ALP: alkaline phosphatase
LDH: lactate dehydrogenase
GAR: γ-glutamyl transferase to albumin ratio
GGT: γ-glutamyl transferase
ALB: albumin
CTCAE: Common Terminology Criteria for Adverse Events
ULN: upper limit of normal.
