# Supplementary material for: A Prognostic Model for Breast Cancer With Liver Metastasis
Source: Front Oncol. 2020 Sep 2;10:1342. doi: 10.3389/fonc.2020.01342 (PMC7493788; doi:10.3389/fonc.2020.01342)
Supplement: Supplementary file 1 [file Data_Sheet_1.docx]

| Table S1.Baseline characteristics of breast cancer with liver metastases (Validation set) | | | | | |
| --- | --- | --- | --- | --- | --- |
|  | Score | | | | Sig |
|  | Total  N= 307 ,n(%) | Low risk  N = 133, n (%) | Intermediate risk N =137 , n (%) | High risk  N =37,n (%) |  |
| Age, years | | | | | |
| <60 | 242(78.8) | 106(79.7) | 108(78.8) | 28(75.7) | 0.887 |
| ≥60 | 65(21.1) | 27(20.3) | 29(21.2) | 9(24.3) |  |
| HR status | | | | | |
| Negative | 142(46.3) | 43(32.3) | 71(51.8) | 28(75.7) | <0.001 |
| Positive | 165(53.7) | 90(67.7) | 66(48.2) | 9(24.3) |  |
| HER2 status | | | | | |
| Negative | 184(59.9) | 57(42.9) | 94(68.6) | 33(89.2) | <0.001 |
| Positive | 123(40.1) | 76(57.1) | 43(31.4) | 4(10.8) |  |
| Surgery of primary site | | | | | |
| NO | 71(23.1) | 47(35.3) | 22(16.1) | 2(5.4) | <0.001 |
| Yes | 236(76.9) | 86(64.7) | 115(83.9) | 35(94.6) |  |
| Prior (neo)adjuvant chemotherapy | | | | | |
| NO | 75(24.4) | 51(38.3) | 22(16.1) | 2(5.4) | <0.001 |
| Yes | 232(75.6) | 82(61.7) | 115(83.9) | 35(94.6) |  |
| MFI, months | | | | | |
| De novo metastatic | 68(22.1) | 47(35.3) | 19(13.9) | 2(5.4) | <0.001 |
| MFI≤24 | 113(36.8) | 28(21.1) | 58(42.3) | 27(73.0) |  |
| MFI>24 | 126(41.0) | 58(43.6) | 60(43.8) | 8(21.6) |  |
| Brain metastases | | | | | |
| NO | 302(98.4) | 132(99.2) | 134(97.8) | 36(97.3) | 0.533 |
| Yes | 5(1.6) | 1(0.8) | 3(2.2) | 1(2.7) |  |
| Lung metastases | | | | | |
| NO | 231(75.2) | 120(90.2) | 93(67.9) | 18(48.6) | <0.001 |
| Yes | 76(24.8) | 13(9.8) | 44(32.1) | 19(51.4) |  |
| Bone metastases | | | | | |
| NO | 170(55.4) | 94(70.7) | 65(47.4) | 11(29.7) | <0.001 |
| Yes | 137(44.6) | 39(29.3) | 72(52.6) | 26(70.3) |  |
| Distant lymph nodes metastases | | | | | |
| NO | 184(59.9) | 96(72.2) | 75(54.7) | 13(35.1) | <0.001 |
| Yes | 123(40.1) | 37(27.8) | 62(45.3) | 24(64.9) |  |
| Liver metastases distribution | | | | | |
| Unilobar | 78(25.4) | 42(31.6) | 32(23.4) | 4(10.8) | 0.027 |
| Bilobar | 229(74.6) | 91(68.4) | 105(76.6) | 33(89.2) |  |
| Number of liver metastases,No | | | | | |
| 1 or2 | 73(23.8) | 42(31.6) | 27(19.7) | 4(10.8) | 0.009 |
| ≥3 | 234(76.2) | 91(68.4) | 110(80.3) | 33(89.2) |  |
| Maximum diameter of liver metastases ,cm | | | | | |
| ≤3 | 173(56.4) | 82(61.7) | 77(56.2) | 14(37.8) | 0.034 |
| >3 | 134(43.6) | 51(38.3) | 60(43.8) | 23(62.2) |  |
| Hb, g/L | | | | | |
| <100 | 16(5.2) | 3(2.3) | 8(5.8) | 5(13.5) | 0.026 |
| ≥100 | 291(94.8) | 130(97.7) | 129(94.2) | 32(86.5) |  |
| Total bilirubin | | | | | |
| ≤1.5 ULN | 300(97.7) | 133(100.0) | 134(97.8) | 33(89.2) | 0.001 |
| >1.5 ULN | 7(2.3) | 0 | 3(2.2) | 4(10.8) |  |
| ALT | | | | | |
| ≤3 ULN | 294(95.8) | 130(97.7) | 132(96.4) | 32(86.5) | 0.022 |
| >3 ULN | 13(4.2) | 3(2.3) | 5(3.6) | 5(13.5) |  |
| AST |  |  |  |  |  |
| ≤3 ULN | 282(91.9) | 128(96.2) | 127(92.7) | 27(73.0) | <0.001 |
| >3 ULN | 25(8.1) | 5(3.8) | 10(7.3) | 10(27.0) |  |
| LDH,U/L | | | | | |
| ≤ 250 | 193(62.9) | 115(86.5) | 74(54.0) | 4(10.8) | <0.001 |
| >250 | 114(37.1) | 18(13.5) | 63(46.0) | 33(89.2) |  |
| ALP | | | | | |
| ≤2.5 ULN | 296(96.4) | 130(97.7) | 135(98.5) | 31(83.8) | 0.001 |
| >2.5 ULN | 11(3.6) | 3(2.3) | 2(1.5) | 6(16.2) |  |
| GAR | | | | | |
| ≤1.5 | 204(66.4) | 115(86.5) | 78(56.9) | 11(29.7) | <0.001 |
| >1.5 | 103(33.6) | 18(13.5) | 59(43.1) | 26(70.3) |  |

NOTE. HR, hormone receptor;HER2, human epidermal growth factor receptor 2; MFI: metastasis-free interval; HB, hemoglobin; ULN, upper limits of normal; ALT, alanine aminotransferase; AST, aspartate aminotransferase; LDH, lactate dehydrogenase; ALP, alkaline phosphatase; GAR, γ-glutamyltransferase to albumin ratio;

| Table S2. Bootstrap for Variables in the equation(Training set) | | | | | |
| --- | --- | --- | --- | --- | --- |
| Variables | B | Bias | SE | Significance | 95% Confidence interval |
| HR | 0.554 | 0.019 | 0.116 | 0.001 | 0.351-0.813 |
| HER2 | 0.480 | 0.015 | 0.112 | 0.001 | 0.270-0.722 |
| MFI≤24( *VS* de novo) | 0.941 | -0.009 | 0.164 | 0.001 | 0.604-1.259 |
| MFI>24( *VS* de novo) | 0.477 | -0.016 | 0.165 | 0.005 | 0.136-0.796 |
| Brain metastases | 0.569 | -0.003 | 0.252 | 0.016 | 0.096-1.084 |
| Lung metastases | 0.270 | 0.000 | 0.110 | 0.014 | 0.051-0.492 |
| Bone metastases | 0.258 | 0.007 | 0.113 | 0.020 | 0.051-0.493 |
| Total bilirubin | 0.833 | 0.058 | 0.489 | 0.084 | 0.003-2.003 |
| LDH | 0.505 | 0.008 | 0.125 | 0.001 | 0.264-0.773 |
| GAR | 0.370 | 0.013 | 0.121 | 0.005 | 0.157-0.627 |

NOTE. B: regression coefficient; SE: standard error; HR, hormone receptor; HER2, human epidermal growth factor receptor 2; MFI: metastasis-free interval; LDH, lactate dehydrogenase; GAR, γ-glutamyltransferase to albumin ratio;

Figure S1.Selection of breast cancer patients with liver metastases

Figure S2.ROC curve of GAR
