# Supplementary figures and images for: A Prognostic Model for Breast Cancer With Liver Metastasis
Source: Front Oncol. 2020 Sep 2;10:1342. doi: 10.3389/fonc.2020.01342 (PMC7493788; doi:10.3389/fonc.2020.01342)

S1.

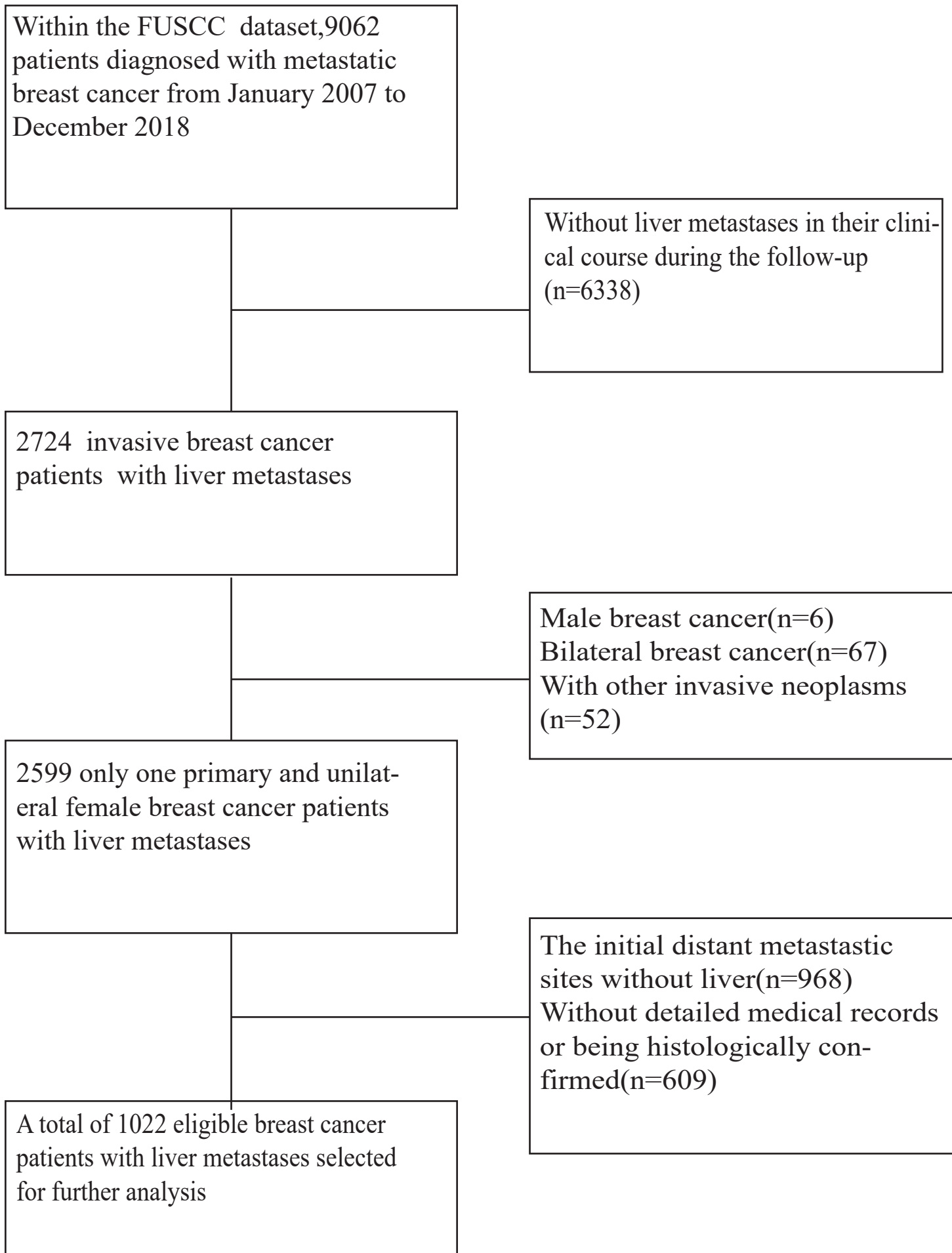

Supplement: Supplementary file 2 [file Image_1.pdf]

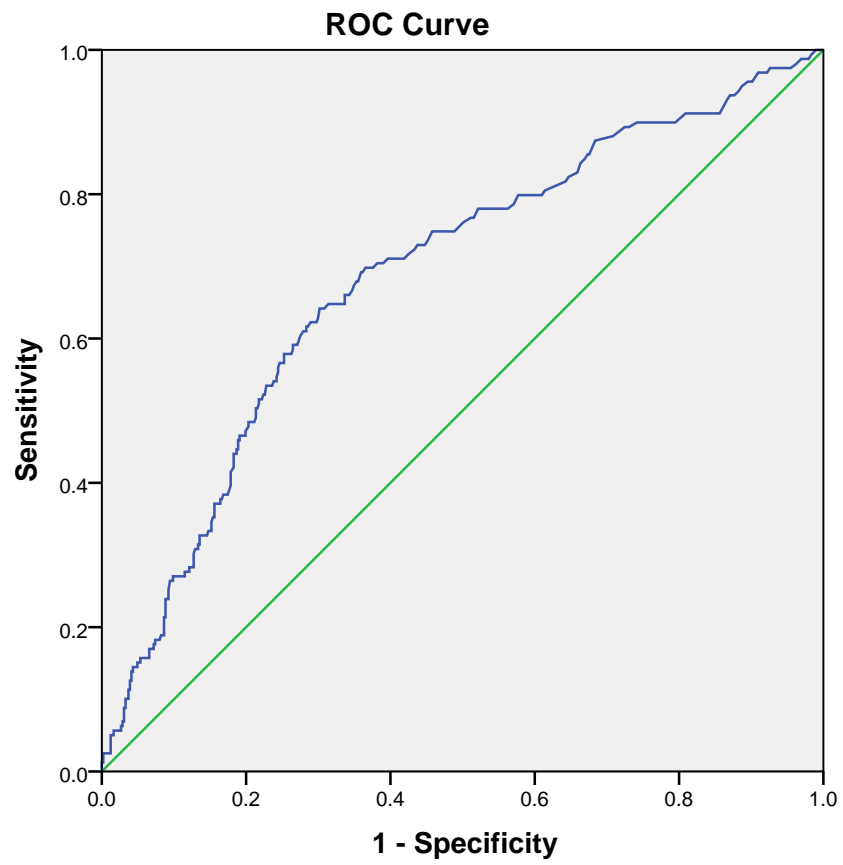

Diagonal segments are produced by ties.

Supplement: Supplementary file 3 [file Image_2.pdf]
